# GABAAR-mediated tonic inhibition differentially modulates intrinsic excitability of VIP- and SST- expressing interneurons in layers 2/3 of the somatosensory cortex

**DOI:** 10.3389/fncel.2023.1270219

**Published:** 2023-10-12

**Authors:** Karolina Bogaj, Roksana Kaplon, Joanna Urban-Ciecko

**Affiliations:** Laboratory of Electrophysiology, Nencki Institute of Experimental Biology, Warsaw, Poland

**Keywords:** barrel cortex, interneurons, tonic inhibition, excitability, GABA, somatostatin interneurons, VIP interneurons

## Abstract

Extrasynaptic GABAA receptors (GABAARs) mediating tonic inhibition are thought to play an important role in the regulation of neuronal excitability. However, little is known about a cell type-specific tonic inhibition in molecularly distinctive types of GABAergic interneurons in the mammalian neocortex. Here, we used whole-cell patch-clamp techniques in brain slices prepared from transgenic mice expressing red fluorescent protein (TdTomato) in vasoactive intestinal polypeptide- or somatostatin- positive interneurons (VIP-INs and SST-INs, respectively) to investigate tonic and phasic GABAAR-mediated inhibition as well as effects of GABAA inhibition on intrinsic excitability of these interneurons in layers 2/3 (L2/3) of the somatosensory (barrel) cortex. We found that tonic inhibition was stronger in VIP-INs compared to SST-INs. Contrary to the literature data, tonic inhibition in SST-INs was comparable to pyramidal (Pyr) neurons. Next, tonic inhibition in both interneuron types was dependent on the activity of delta subunit-containing GABAARs. Finally, the GABAAR activity decreased intrinsic excitability of VIP-INs but not SST-INs. Altogether, our data indicate that GABAAR-mediated inhibition modulates neocortical interneurons in a type-specific manner. In contrast to L2/3 VIP-INs, intrinsic excitability of L2/3 SST-INs is immune to the GABAAR-mediated inhibition.

## Introduction

GABA (gamma-aminobutyric acid) is the main inhibitory neurotransmitter in the cerebral cortex and plays an essential role in the regulation of the neuronal activity through two types of receptors (GABAARs and GABABRs). Both classes of receptors evoke two forms of inhibition: phasic and tonic (Lee and Maguire, 2014). Phasic inhibition is mediated by GABAARs and GABABRs located within the postsynaptic and perisynaptic membrane, whereas tonic inhibition is mediated by the extrasynaptic receptors (Glykys and Mody, 2007). It has been shown that tonic inhibition might be activated by ambient GABA that diffuses throughout the extracellular space and binds to the extrasynaptic GABAA and GABABRs (Farrant and Nusser, 2005). GABAARs mediating tonic inhibition are composed of unique subunit types (Belelli et al., 2009). Most extrasynaptic GABAARs contain alpha 5, alpha 4 or delta subunits (Belelli et al., 2009). GABAARs with these subunits display a high affinity for GABA and a low efficacy (Saxena and Macdonald, 1994). Extrasynaptic GABAARs also are weakly desensitizing (Saxena and Macdonald, 1994). These features allow detection of micromolar concentrations of ambient neurotransmitter and enable high potential for allosteric modulation. Cell-type specific expression of specific GABAAR subunits mediating tonic inhibition in particular neuronal types are still under investigation.

GABAAR-mediated inhibition can differentially modulate neuronal excitability (Bryson et al., 2020). However, the role of tonic inhibition in specific neuronal types is still not fully understood. The effect of GABA on neuronal excitability is determined by the reversal potential for Cl^−^, which is the major ion mediating the GABAAR current. In general, in adult neurons, the reversal potential for Cl^−^ is set close to the resting membrane potential. However, the resting membrane potential and the reversal potential for Cl^−^ vary among cell types, therefore, the effect of GABA can be either hyperpolarizing or depolarizing (Hausselt et al., 2007; Khirug et al., 2008). Previous works have shown that tonic inhibition decreases the excitability of Pyr cell by increasing rheobase (Mitchell and Silver, 2003; Pavlov et al., 2009; Silver, 2010). The effect of tonic inhibition on interneuron excitability varies depending on the interneuron type (Song et al., 2011; Pavlov et al., 2014; Bryson et al., 2020). Depolarizing effect of GABA has been found in fast spiking (FS) CA3 stratum lucidum interneurons (Pavlov et al., 2014) and in other interneurons in the cerebellum (Chavas and Marty, 2003), amygdala (Woodruff et al., 2006) and striatum (Bracci and Panzeri, 2006). Studies using biophysically detailed neuron models have predicted that effects of tonic inhibition on interneuron excitability is determined by the variation in the electrophysiological properties of specific interneurons (Pavlov et al., 2014).

GABAergic interneurons form a broad spectrum of subtypes based on the morphology, electrophysiological properties, the expression of characteristic molecular markers and specific functions in the network (Rudy et al., 2011). Two types of the GABAergic interneurons (VIP- and SST-INs) are involved in a disinhibitory loop in the neocortex (Pfeffer et al., 2013; Jiang et al., 2015, 2023). Namely, L2/3 VIP-INs inhibit L2/3 SST-INs and in turn release Pyr neurons from SST-IN-mediated inhibition. Such a disinhibitory effect has been found to be essential in learning process (Lee et al., 2013). However, VIP-INs also are reciprocally inhibited by SST-INs (Jiang et al., 2015). Thus, L2/3 VIP- and SST-INs play specific roles in the neocortex. Both interneuron types possess specific electrophysiological properties and various firing phenotypes (Prönneke et al., 2015, 2019; Liguz-Lecznar et al., 2016; Jiang et al., 2023) and can be further divided into many subgroups depending on the molecular, electrophysiological, morphological or functional features (Rudy et al., 2011; Liguz-Lecznar et al., 2016; Urban-Ciecko and Barth, 2016; Gouwens et al., 2020; Hostetler et al., 2023; Jiang et al., 2023; Munguba et al., 2023).

Previous studies have shown no tonic inhibition in L2/3 SST-INs in the mouse frontoparietal cortex (Vardya et al., 2008) and a weak tonic inhibition in L2/3 SST-INs in the barrel cortex (Donato et al., 2023). However, in these studies, transgenic “GIN” mice or X98 mouse line that express enhanced green florescent protein in a subset of SST-INs were used (Oliva et al., 2000). Other studies, using Sst-Cre mouse line have revealed a weak tonic inhibition in hippocampal SST-INs (Huang et al., 2023; Wyroślak et al., 2023). To our best knowledge, tonic inhibition has not yet been studied in VIP-INs.

Here, we used whole-cell patch-clamp techniques in brain slices prepared from transgenic mice expressing red fluorescent protein (TdTomato) in VIP- or SST-INs and investigated tonic and phasic GABAAR-mediated inhibition in these interneurons in layers 2/3 of the somatosensory (barrel) cortex. Then we analyzed the effect of GABAAR-mediated inhibition on their intrinsic excitability.

We found that L2/3 VIP- and SST-INs of the barrel cortex displayed tonic GABAAR inhibition at different levels – higher in VIP- than in SST-INs. In both interneuron types, tonic inhibition was mediated by delta subunit-containing GABAARs. GABAARs modulated intrinsic excitability in an interneuron type-specific manner. The GABAAR activity decreased intrinsic excitability of VIP-INs but had no effect on the excitability of SST-INs. Altogether, our study reveals that molecularly distinct interneurons with the very similar firing phenotypes are differentially modulated by tonic GABAAR-mediated inhibition.

## Materials and methods

### Ethical approval

All procedures were conducted in accordance with the Act on the Protection of Animals Used for Scientific or Educational Purposes in Poland (Act of 15 January 2015, changed 17 November 2021; directive 2010/63/EU) and were approved by Polish Ministry of Environment (Dec. No 47/2019). The study was reported in agreement with ARRIVE guidelines.

### Animals

The following strains of mice were used: (1) Sst-IRES-Cre (Jackson Labs stock #013044); (2) Vip-IRES-Cre (Jackson Labs stock #010908); (3) Ai14 mice (Jackson Labs stock #007908). Experiments were performed in offspring of Sst-IRES-Cre or Vip-IRES-Cre crossed to Ai14 (floxed-Tdt) reporter mice; Sst-Cre::Ai14 mice and VIP-Cre::Ai14 mice, respectively. All transgenes were used as heterozygotes and both sexes were used. Mice were housed under controlled light cycles (12-h light–dark cycles) with an *ad libitum* access to food and water.

### Brain slice preparation

At the age of P18–P30, where P0 indicates the day of birth, mice were anaesthetized with isoflurane and killed by decapitation using the procedure in accordance with the Polish Animal Protection Act (Act of 15 January 2015, changed 17 November 2021; directive 2010/63/EU).

Brain slices (350 μm thick) were cut in an “across-row” procedure in which the anterior end of the brain was cut along a 45° plane toward the midline (Finnerty et al., 1999). Slices were cut at 2–4°C, then recovered and maintained at 32°C in artificial cerebrospinal fluid (ACSF) composed of (in mM): 113 NaCl, 2.5 KCl, 1.3 MgSO_4_, 2.5 CaCl_2_, 1 NaH_2_PO_4_, 26.2 NaHCO_3_, 11 glucose equilibrated with 95/5% O_2_/CO_2_. Recordings were performed at 32°C in ACSF of the same composition as above.

### Whole-cell recording

Somata of L2/3 neurons in the somatosensory (barrel) cortex were targeted for whole-cell recordings. Neurons were classified as Pyr neurons according to Pyr-like soma shape, the presence of an apical dendrite, as well as based on regular spiking pattern in response to 500 ms suprathreshold intracellular current injection. SST-INs and VIP-INs were identified using fluorescent reporter gene expression in Sst-Cre::Ai14 and Vip-Cre::Ai14 mice, respectively. For analysis of tonic GABAAR inhibition, a high [Cl^−^] internal solution was used (in mM): 140 KCl, 1 MgCl_2_, 10 HEPES, 0.5 EGTA, 4 Mg-ATP, pH 7.25–7.35, 290 mOsm (Urban-Ciecko et al., 2010; Urban-Ciecko and Mozrzymas, 2011). For study of intrinsic excitability, a low [Cl^−^] internal solution was composed of (in mM): 125 potassium gluconate, 2 KCl, 10 HEPES, 0.5 EGTA, 4 Mg-ATP, and 0.3 Na-GTP, at pH 7.25–7.35 and 290 mOsm (Urban-Ciecko et al., 2015, 2018). For recordings of sIPSCs, the internal solution contained (in mM): 130 cesium gluconate, 10 HEPES, 0.5 EGTA, 8 NaCl, 10 tetraethylammonium chloride, 5 QX-314, 4 Mg-ATP, and 0.3 Na-GTP, at pH 7.25–7.35, 290 mOsm (Kanigowski et al., 2023). Patch electrodes were made from borosilicate glass and had 2–4 MOhm or 4–6 MOhm when filled with the high [Cl^−^] internal solution and the low [Cl^−^] solution, respectively.

Electrophysiological data were acquired by Multiclamp 700B (Molecular Devices) and digitized with Digidata 1550B (Molecular Devices). The data were filtered at 3 kHz, digitized at 20 kHz and collected by pClamp (Molecular Devices). Series and input resistances were analyzed online, recordings were discarded when access or series resistances were unstable more than 20%.

Membrane parameters were measured every 10 s using a 100 ms-pulse of −10 mV and − 10 pA in voltage- and current-mode, respectively.

Tonic currents were recorded in voltage-clamp mode at the holding potential of −70 mV. The value of the tonic current was calculated as the baseline shift after the application of drugs and normalized to the whole-cell membrane capacitance (the current density). The baseline (holding) current was measured every 10 s and the baseline shift was calculated as a change in the holding current after 10 min with the appropriate drug. The current noise was measured as the standard deviation (SD) of the holding current (Bright and Smart, 2013).

Spontaneous inhibitory postsynaptic currents (sIPSCs) were recorded in voltage-clamp at 0 mV in the cesium gluconate-based internal solution.

Intrinsic excitability was assessed using square pulses of 500 ms of an amplitude increasing up to maximal firing frequency (steps of 10 pA). To control for potential effects of GABAAR agents on the resting membrane potential, the membrane potential of interneurons was maintained at −70 mV across different pharmacological conditions. For intrinsic excitability, a curve of the relation between the frequency of action potentials and the intensity of the injected current (F-I curve) was plotted for every neuron in control ACSF and after the drug application. The effect of the drug on excitability was analyzed as the difference in the rheobase, the maximal firing frequency and the difference of the spiking frequency at the same current steps in the comparison to control ACSF. The rheobase means a step of the injected current at the minimal intensity that evoked at least one spike.

### Pharmacology

The GABAAR blocker picrotoxin (PTX, 100 μM) and a delta subunit-containing GABAAR superagonist (4,5,6,7- tetrahydroisoxazolo[5,4-c]pyridin-3-ol, THIP, 20 μM) were bath applied for at least 10 min to assess drug effects. All the pharmacological agents were purchased from Tocris.

### Data analysis

Population data are presented as mean ± SD. One cell in a slice and 1–3 cells were analyzed in an individual mouse. The normality distribution was tested with the Shapiro–Wilk test and equal variance was analyzed with Brown-Forsythe test. For comparison of multiple groups, a one way ANOVA (parametric) was used, followed by a Tukey *post hoc* test, or a Kruskal Wallis test (nonparametric) was used, followed by a Dunn’s test. For comparison of two variables in multiple groups a two way ANOVA was used with Holm-Sidak’s test. To compare an effect of a drug within a cell, a two-tailed paired *t*-test or Wilcoxon test were used depending on the normality distribution. Statistical significance was defined as *p < 0.05, **p ≤ 0.01, and ***p ≤ 0.001.

## Results

### Diverse firing properties of L2/3 VIP- and SST-INs

**Figure 1:**
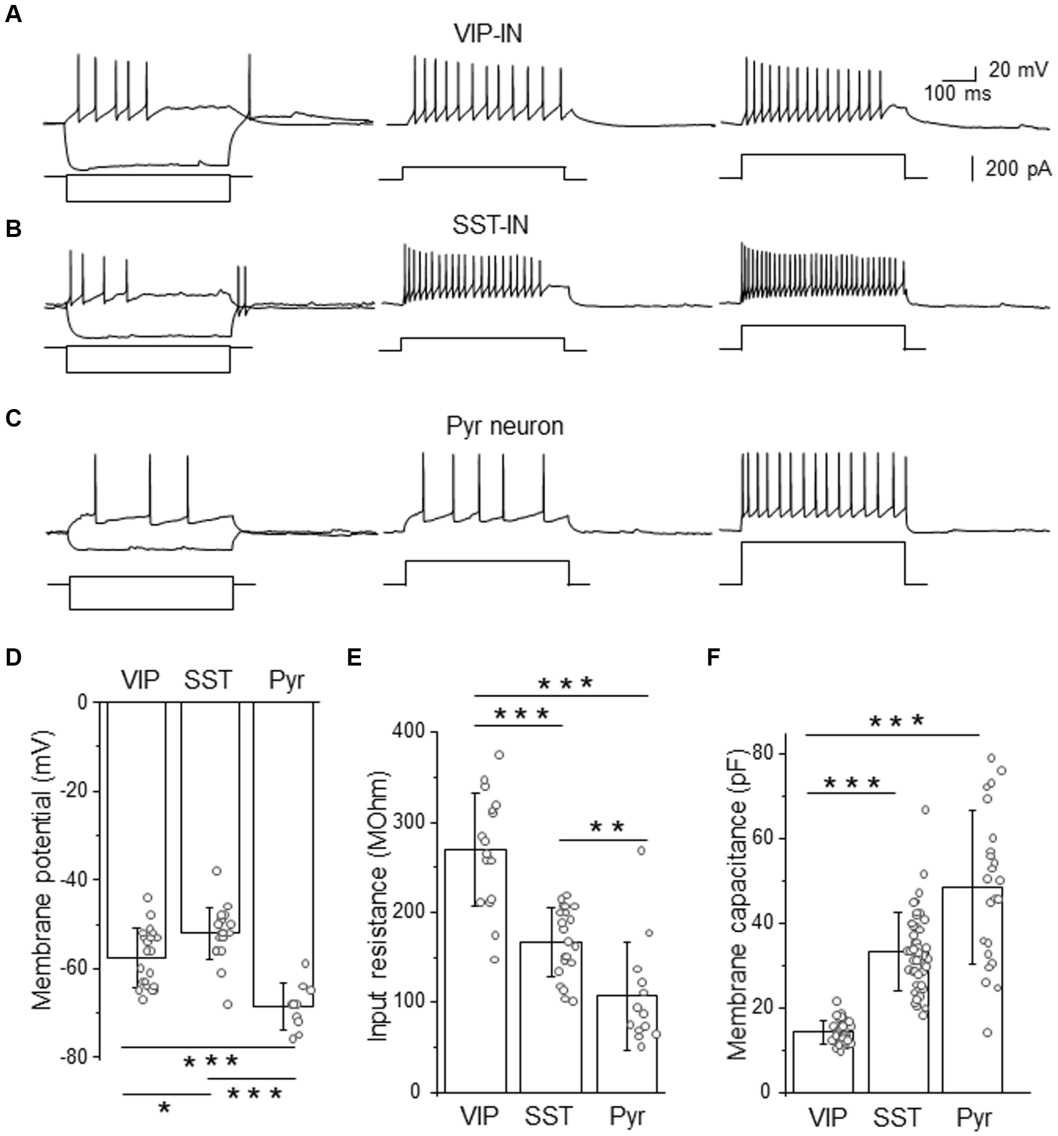
Firing phenotypes of L2/3 VIP- and SST-INs are very similar in a high [Cl^−^]-based internal solution. **(A)** Example traces of VIP-IN firing responses after the somatic current injection of a 500 ms-long pulse at the intensity of; 20 pA and −200 pA (*left traces*); 100 pA (*middle trace*) and 200 pA (*right trace*). Current steps shown below firing traces. **(B)** The same as in **(A)** but for SST-IN. **(C)** The same as in **(A)** but for a pyramidal (Pyr) neuron. **(D)** The comparison of mean (±SD) resting membrane potentials in 3 types of neurons. **(E)** The same as in **(D)** but for the input resistance. **(F)** The same as in **(D)** but for the membrane capacitance. *p < 0.05, **p ≤ 0.01, and ***p ≤ 0.001.

To study different types of GABAergic interneurons, we used transgenic mice with fluorescently labeled interneurons. Before the analysis of tonic current, we checked firing phenotypes of fluorescently labeled interneurons because we were interested whether L2/3 VIP- and SST-INs might be differentiated according to their firing patterns in the high [Cl^−^] internal solution used for the analysis of tonic inhibition. We found that both L2/3 VIP- and SST-INs exhibit a variety of firing patterns (Figure 1 and Supplementary Figure 1) in this condition. According to Petilla terminology (Petilla Interneuron Nomenclature Group, 2008), both types of interneurons displayed accommodating firing (AC), low-threshold spiking (LTS) firing patterns with rebound spiking after hyperpolarizing current step (Figures 1A,B and Supplementary Figures 1A,B) or irregular spiking (IR). A part of SST-INs showed fast spiking (FS) patterns (Supplementary Figure 1B). In contrast to the recordings in the low [Cl^−^] internal solution, there were no L2/3 VIP-INs with the burst firing in the high [Cl^−^] internal solution (Supplementary Figure 1A). These results indicate that different recording conditions might influence the activity of particular channels and thus the overall properties of the firing phenotypes. Finally, putative Pyr neurons responded with a regular spiking phenotype without rebound spikes (Figure 1C).

Analysis of electrophysiological properties such as the resting membrane potential (Figure 1D), the input resistance (Figure 1E) and the membrane capacitance (Figure 1F) revealed significant differences between VIP- and SST-INs and Pyr cells. In agreement with the literature data (Gentet et al., 2010, 2012; Urban-Ciecko et al., 2015; Dobrzanski et al., 2022; Kanigowski et al., 2023) interneurons exhibited more depolarized resting membrane potential than Pyr cells. SST-INs had the most depolarized resting membrane potentials (−52.00 ± 5.84 mV, *n* = 21 cells in 21 mice) compared to VIP-INs (−57.57 ± 6.61 mV, *n* = 21 cells in 19 mice) and Pyr neurons (−68.60 ± 5.17 mV, *n* = 10 cells in 8 mice, *p* = 0.012 for VIP-INs versus SST-INs, *p* < 0.001 for VIP-INs and SST-INs versus Pyr, one way ANOVA with Tukey test, Figure 1D). The input resistance was the highest in VIP-INs (268.98 ± 62.60 MOhm, *n* = 17 cells in 17 mice) in comparison to SST-INs (167.33 ± 38.39 MOhm, *n* = 21 cells in 21 mice, *p* < 0.001, one way ANOVA with Tukey test, Figure 1E) and Pyr (107.15 ± 60.09 MOhm, *n* = 13 cells in 7 mice, *p* < 0.001), also SST-INs had higher input resistance than Pyr cells (*p* = 0.007). In contrast, the whole-cell membrane capacitance was the lowest in VIP-INs (14.32 ± 2.70 pF, *n* = 38 cells in 19 mice) compared to SST-INs (33.40 ± 9.14 pF, *n* = 49 cells in 21 mice) and Pyr (48.61 ± 18.06 pF, *n* = 23 cells in 15 mice, *p* < 0.001, Kruskal-Wallis with Dunn’s tests, Figure 1F), whereas SST and Pyr did not differ in the membrane capacitance (*p* = 0.07).

Summarizing, analysis of GABAergic interneurons according to their firing patterns would not discriminate between molecularly distinct interneurons, even though they have statistically different basic electrophysiological properties of the membrane. It is worthwhile to mention that an LTS firing phenotype had been assumed to be characteristic for SST-INs in previous studies before the era of transgenic tools, for rev (Liguz-Lecznar et al., 2016). Here, we show that this assumption is not accurate.

### L2/3 VIP-INs exhibit stronger tonic GABAAR-mediated inhibition than SST-INs

**Figure 2:**
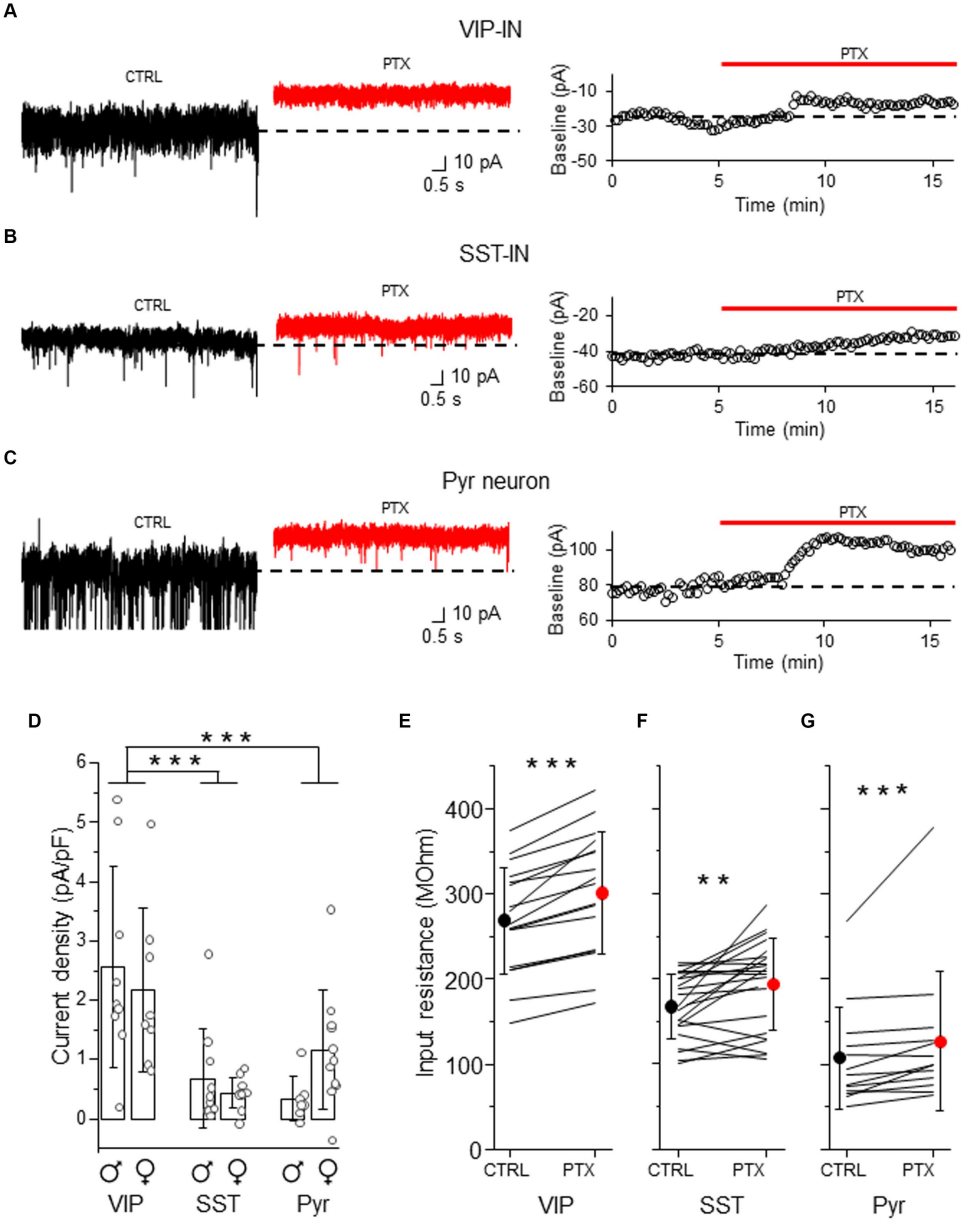
Tonic GABAAR-mediated inhibition is stronger in L2/3 VIP-INs than in SST-INs. **(A)** Example traces of current recordings in control (CTRL) ACSF and after picrotoxin (PTX) application in VIP-IN (*left*), the baseline holding current measured every 10 s and plotted against time was used for the estimation of the tonic current (*right*). **(B)** The same as in **(A)** but for SST-IN. **(C)** The same as in **(A)** but for a pyramidal (Pyr) neuron. **(D)** The comparison of mean (±SD) tonic current density in the presence of the GABAAR blocker (PTX) in VIP-, SST-INs and Pyr neurons. **(E)** With-in cell comparison and mean (±SD) input resistance of VIP-INs in CTRL and after PTX. **(F)** The same as in **(E)** but for SST-INs. **(G)** The same as in **(E)** but for Pyr neurons. *p < 0.05, **p ≤ 0.01, and ***p ≤ 0.001.

Because tonic GABAAR inhibition has previously been studied in Pyr neurons (Drasbek and Jensen, 2006; Vardya et al., 2008; Urban-Ciecko et al., 2010), we used these cells for the comparison to assess tonic inhibition in distinct interneuron types (Figure 2). After bath application of the GABAAR blocker (PTX), we observed a shift in baseline current and a reduction in the current noise in both interneuron types (Figures 2A,B and Supplementary Figures 2A,B) and in Pyr (Figure 2C and Supplementary Figure 2C). The current density was larger in VIP-INs (2.56 ± 1.69 pA/pF, *n* = 9 cells in 4 males and 2.17 ± 1.38 pA/pF, *n* = 8 cells in 5 females, Figure 2D) than in SST-INs (0.68 ± 0.84 pA/pF, *n* = 10 cells in 6 males and 0.43 ± 0.26 pA/pF, *n* = 11 cells in 6 females, *p* < 0.001, two way ANOVA with Holm Sidak’s test) and Pyr (0.34 ± 0.38 pA/pF, *n* = 7 cells in 5 males and 1.17 ± 1.00 pA/pF, *n* = 11 cells in 6 females, *p* < 0.001), whereas the current density in SST-INs was similar to Pyr cells (*p* = 0.556, Figure 2D). Because the strength of tonic inhibition might be sex-dependent (Urban-Ciecko and Mozrzymas, 2011), we compared the values of the current density between males and females, and we found no differences in tonic inhibition in these 3 types of neurons in regards to the sex of animals (*p* = 0.817, two way ANOVA with Holm Sidak’s test, Figure 2D). Furthermore, we observed that the application of PTX increased the input resistance in each neuronal type (for VIP-INs from 268.98 ± 62.60 MOhm in CTRL to 301.05 ± 72.13 MOhm in PTX, *n* = 21 cells in 19 mice, *p* < 0.001, Wilcoxon test, Figure 2E; for SST-INs from 167.33 ± 38.39 MOhm in CTRL to 193.05 ± 54.28 MOhm in PTX, *n* = 17 cells in 17 mice, *p* = 0.004, two-tailed paired *t*-test, Figure 2F; for Pyr cells from 107.15 ± 60.09 MOhm in CTRL to 126.77 ± 82.36 MOhm in PTX, *n* = 13 cells in 7 mice, *p* < 0.001, Wilcoxon test, Figure 2G). Because there were no differences in tonic inhibition concerning the sex of mice (Figure 2D), we pooled data from males and females for the analysis of the input resistance (Figures 2E–G).

Altogether, our results indicate that GABAARs influence membrane properties of both interneuron types and Pyr cells in a way that would promote a decrease of neuronal excitability.

### Tonic inhibition in L2/3 VIP- and SST-INs is mediated by delta subunit-containing GABAARs

**Figure 3:**
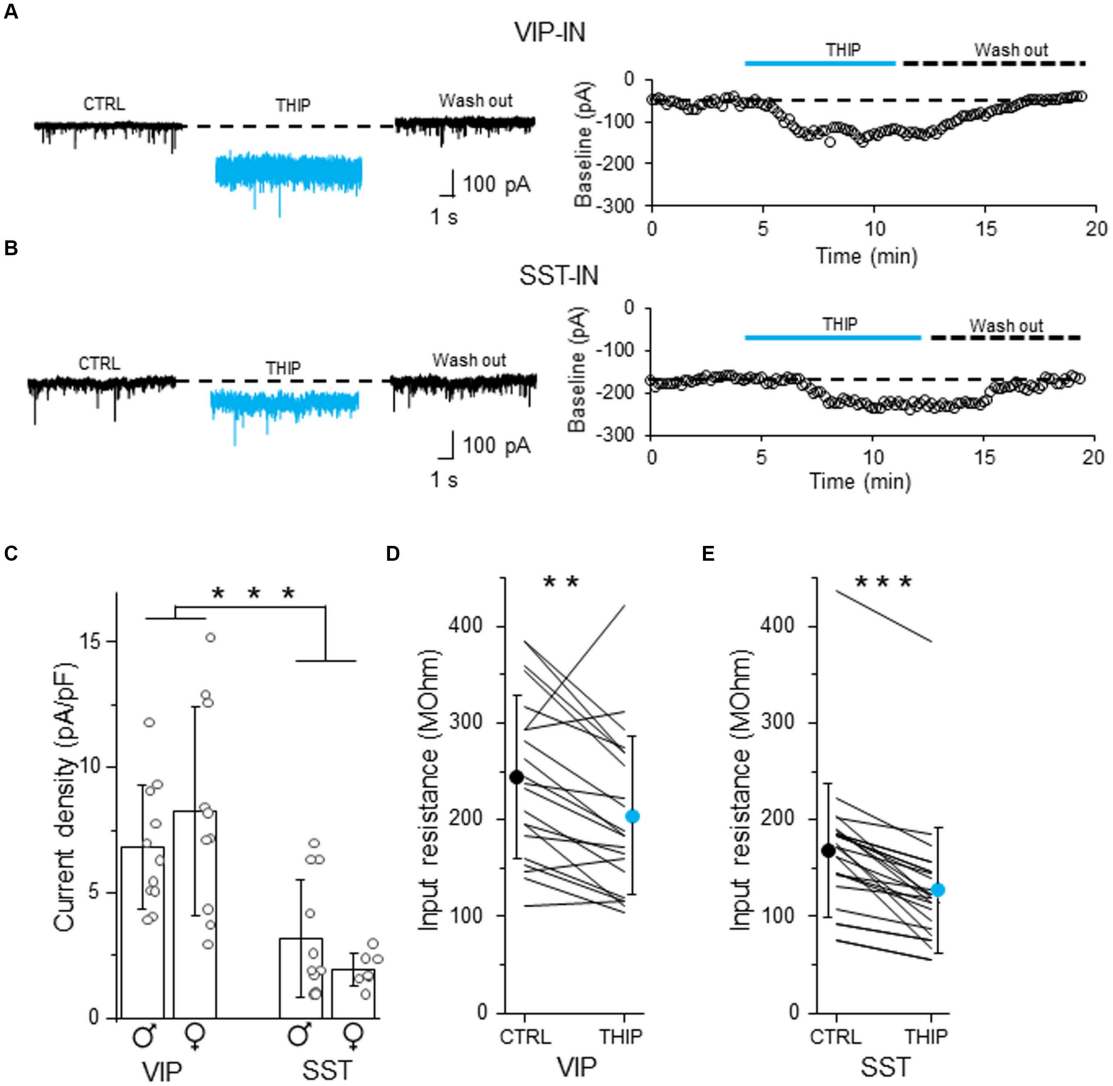
Tonic inhibition in L2/3 VIP- and SST-INs depends on delta subunit-containing GABAARs. **(A)** Example traces of current recording in control (CTRL) ACSF, after the application of a selective GABAAR agonist with a preference for delta subunit-containing GABAARs (4,5,6,7-tetrahydroisoxazolo[5,4-c]pyridin-3-ol, THIP) and wash-out condition (*left*), the baseline holding current measured every 10 s and plotted against time was used for the estimation of the tonic current in VIP-IN (*right*). **(B)** The same as in **(A)** but for SST-IN. **(C)** The comparison of mean (±SD) tonic current density in the presence of THIP in VIP- and SST-INs. **(D)** With-in cell comparison and mean (±SD) input resistance of VIP-INs in CTRL and after THIP. **(E)** The same as in **(D)** but for SST-INs. *p < 0.05, **p ≤ 0.01, and ***p ≤ 0.001.

To check whether tonic inhibition in L2/3 VIP- and SST-INs is mediated by delta subunit-containing GABAARs, we bath applied THIP which is a selective GABAAR agonist with a preference for delta subunit-containing GABAARs (Hansen et al., 2004). THIP induced a baseline shift and an increase in the current noise in both VIP- (Figure 3A and Supplementary Figure 3A) and SST-INs (Figure 3B and Supplementary Figure 3B). The effect was reversible after wash out (Figures 3A,B). The current density evoked by THIP was larger in VIP-INs (6.80 ± 2.48 pA/pF, *n* = 11 cells in 5 males and 8.26 ± 4.15 pA/pF, *n* = 10 cells in 5 females, Figure 3C) than in SST-INs (3.16 ± 2.36 pA/pF, *n* = 11 cells in 5 males and 1.94 ± 0.67 pA/pF, *n* = 7 cells in 6 females, *p* < 0.001, two way ANOVA with Holm Sidak’s test) and there was no difference between males and females (*p* = 0.831, two way ANOVA with Holm Sidak’s test). Moreover, THIP reduced the input resistance in both types of the interneurons (for VIP-INs from 244.48 ± 83.89 MOhm in CTRL to 204.43 ± 81.66 MOhm in THIP, *n* = 21 cells in 10 mice, *p* = 0.003, Wilcoxon test, Figure 3D; for SST-INs from 168.72 ± 69.29 MOhm in CTRL to 126.92 ± 64.75 MOhm in THIP, *n* = 25 cells in 21 mice, *p* < 0.001, Wilcoxon test, Figure 3E). Because there were no differences in tonic inhibition in regards to the sex of animals (Figure 3C), we pooled data from males and females for the analysis of the input resistance (Figures 3D,E).

Our data indicate that L2/3 VIP- and SST-INs possess different levels of tonic inhibition mediated by delta subunit-containing GABAARs.

### L2/3 VIP-INs have weaker phasic GABAAR-mediated inhibition than SST-INs

**Figure 4:**
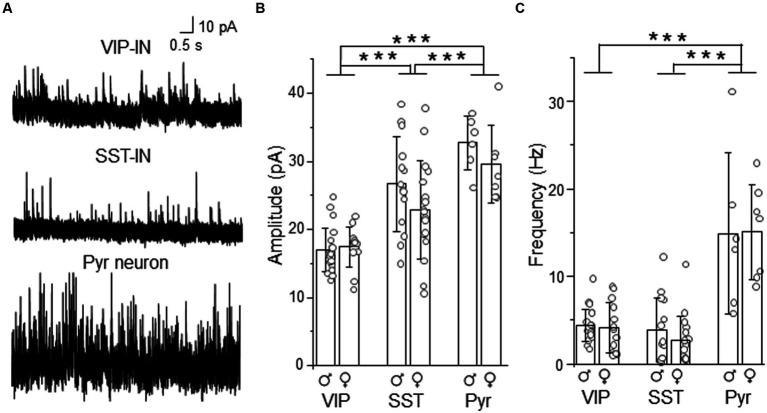
Synaptic (phasic) inhibition is weaker in L2/3 VIP-INs than in SST-INs. **(A)** Example traces of sIPSC recordings in VIP-, SST-IN and Pyr. **(B)** The mean (±SD) amplitude of sIPSCs recorded in these neurons. **(C)** The same as in **(B)** but for the mean (±SD) frequency. *p < 0.05, **p ≤ 0.01, and ***p ≤ 0.001.

To compare whether differences in tonic inhibition of L2/3 SST- and VIP-INs are also accompanied by differences in synaptic (phasic) GABAergic inhibition, we recorded sIPSCs in these interneurons. For better comparison, in some cases recordings were performed in an interneuron and a neighboring Pyr cell within the same slice (Figure 4). We observed that the mean amplitude of sIPSCs was smaller in VIP-INs (16.99 ± 3.20 pA, *n* = 21 cells in 10 males and 17.46 ± 2.94 pA, *n* = 13 cells in 9 females, Figure 4B) compared to SST-INs (26.69 ± 7.01 pA, *n* = 14 cells in 12 males and 22.91 ± 7.24 pA, *n* = 17 cells in 13 females) and Pyr (32.72 ± 4.02 pA, *n* = 6 cells in 6 males and 29.54 ± 5.71 pA, *n* = 7 cells in 6 females, *p* < 0.001, two way ANOVA with Holm Sidak’s test). Also, sIPSC amplitude was smaller in SST-INs than in Pyr cells (*p* < 0.001, two way ANOVA with Holm Sidak’s test). In case of sIPSC frequency (Figure 4C), there was no difference between VIP-INs (4.40 ± 1.84 Hz, *n* = 21 cells in 10 males and 4.17 ± 2.90 Hz, *n* = 13 cells in 9 females) and SST-INs (3.88 ± 3.72 Hz, *n* = 14 cells in 12 males and 2.70 ± 2.79 Hz, *n* = 17 cells in 13 females, *p* = 0.51, two way ANOVA with Holm Sidak’s test). However, Pyr cells exhibited the highest sIPSC frequency (14.91 ± 9.20 Hz, *n* = 6 cells in 6 males and 15.13 ± 5.43 Hz, *n* = 7 cells in 6 females, *p* < 0.001, two way ANOVA with Holm Sidak’s test). Finally, there were no differences between males and females regarding the amplitude and the frequency of sIPSCs (*p* = 0.092 for the amplitude and *p* = 0.525 for the frequency, two way ANOVA with Holm Sidak’s test).

Summarizing, the analysis showed larger sIPSC amplitude in SST-INs than in VIP-INs but no difference in the frequency of sIPSCs. Taking into account the assumption that the event amplitude is shaped by the receptor number and the receptor subunit composition whereas the frequency of events depends on the number of synaptic inputs (Glasgow et al., 2019), our results suggest that these interneuron types might have different number of synaptic GABAARs or subunit compositions of these receptors but comparable numbers of inhibitory synaptic inputs.

### Intrinsic excitability of L2/3 VIP-INs is controlled by GABAARs

**Figure 5:**
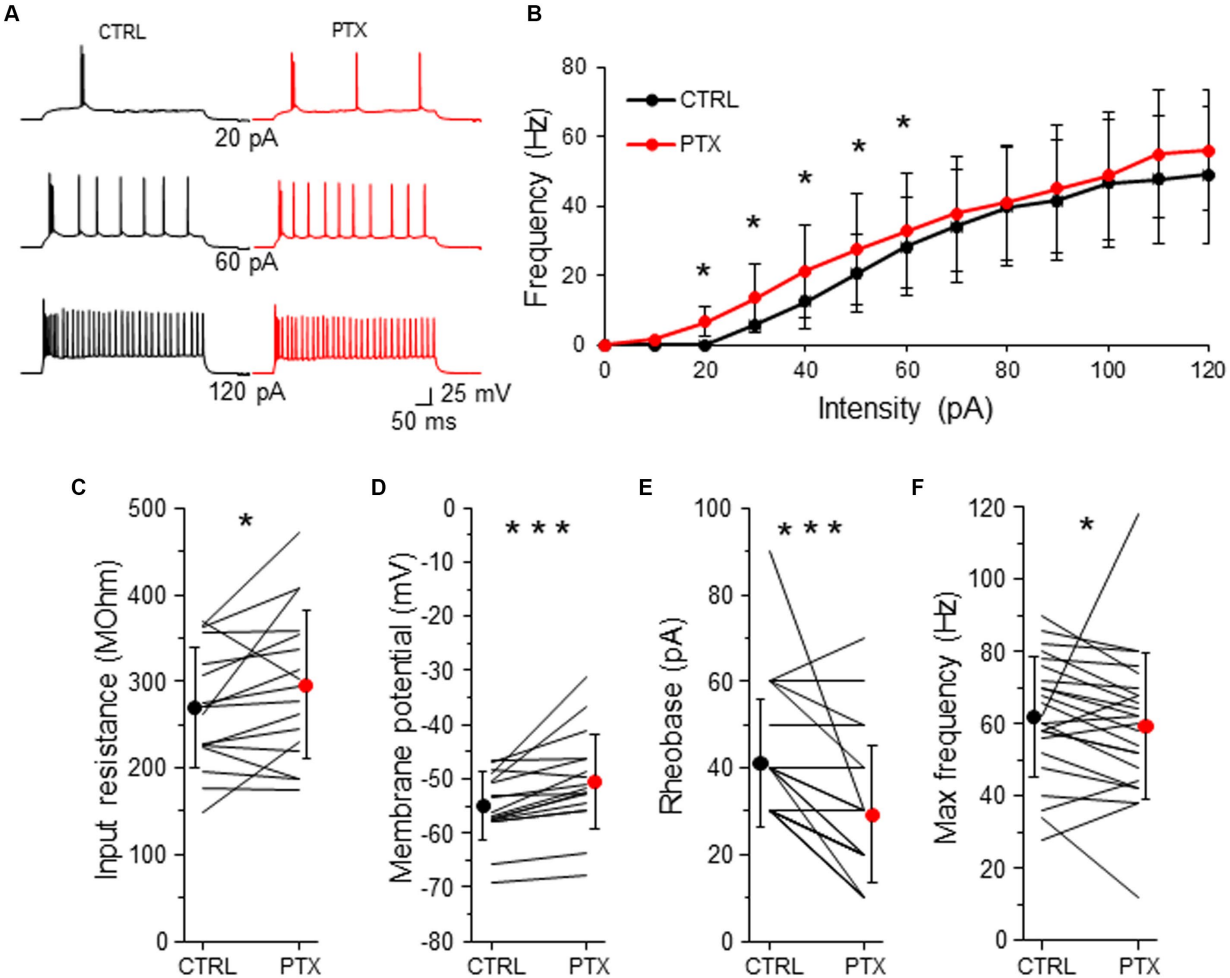
GABAARs decrease intrinsic excitability of L2/3 VIP-INs. **(A)** Example traces of VIP-IN firing responses after the somatic current injection of a 500 ms-long pulse at 3 different intensities (20, 60, 120 pA) in CTRL ACSF (*black traces*) and after PTX application (*red traces*). **(B)** Summary plot of the mean firing frequency (±SD) in response to current injections (from 0 to 120 pA) from VIP-INs recorded in control ACSF and after PTX. **(C)** With-in cell comparison and mean (±SD) input resistance under baseline condition (CTRL) and in the presence of PTX. **(D)** The same as in **(C)** but for membrane potential. **(E)** The same as in **(C)** but for rheobase. **(F)** The same as in **(C)** but for maximal frequency. *p < 0.05, **p ≤ 0.01, and ***p ≤ 0.001.

Tonic inhibition is thought to regulate neuronal excitability, however, the effect might be cell type-specific (Bryson et al., 2020). To study the effect of GABAergic inhibition on intrinsic excitability of L2/3 VIP-INs, we used a canonical internal solution, the K-gluconate-based internal solution with a low [Cl^−^] concentration. In this condition, VIP-INs displayed a variety of the firing patterns (Supplementary Figure 1A), such as burst spiking (Figure 5A), irregular spiking or continuous adapting according to the terminology published in Jiang et al. (2023).

To investigate the impact of GABAergic inhibition upon VIP-IN excitability, we created a curve of the relation between the frequency of action potentials (AP) and the intensity of the injected current (F-I curve) for every neuron in control ACSF and after the application of PTX (Figure 5B). Because there were no differences in phasic and tonic GABAAR-mediated inhibition in respect to the sex of animals (Figures 2–4), we pooled data from males and females for the analysis of neuronal excitability. We observed that PTX shifted the F-I curve to higher AP frequency in lower current intensities (*n* = 25 cells in 13 mice, *p* < 0.05, two-tailed paired *t*-test, Figures 5A,B). Also, PTX application increased the input resistance from 269.79 ± 68.76 MOhm to 296.19 ± 85.86 MOhm (*n* = 17 cells in 13 mice, *p* = 0.049, two-tailed paired t-test, Figure 5C), depolarized the resting membrane potential from −54.94 ± 6.23 mV to −50.48 ± 8.84 mV (*n* = 17 cells in 13 mice, *p* < 0.001, Wilcoxon test, Figure 5D) and decreased the rheobase from 41.20 ± 14.81 pA to 29.20 ± 15.79 pA (*n* = 25 cells in 13 mice, *p* < 0.001, Wilcoxon test, Figure 5E) in VIP-INs. However, the maximal frequency of APs was slightly reduced after PTX (from 62.00 ± 16.56 Hz in CTLR to 59.50 ± 20.20 Hz in PTX, n = 25 cells in 13, *p* = 0.038, Wilcoxon test, Figure 5F).

Taking into account that the rheobase might be the best predictor of the changes in the intrinsic excitability (Bryson et al., 2020), our data show that GABAergic (presumably mostly tonic) inhibition decreases the excitability of L2/3 VIP-INs.

### Intrinsic excitability of SST-INs is immune to the activity of GABAARs

**Figure 6:**
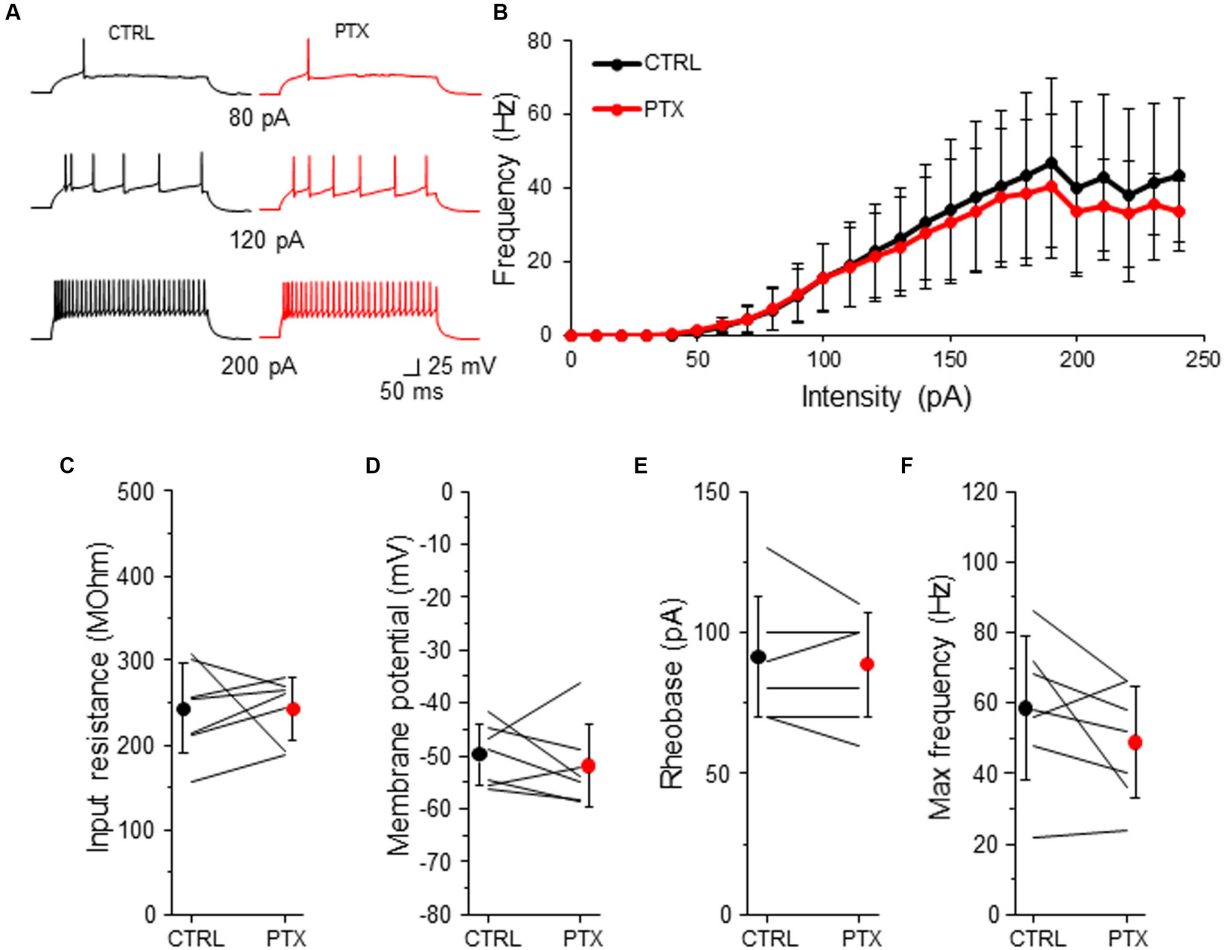
GABAARs has no impact on intrinsic excitability of SST-INs. **(A)** Example traces of SST-IN firing responses after the somatic current injection of a 500 ms-long pulse at 3 different intensities (80, 120, 200 pA) in CTRL ACSF (*black traces*) and after PTX application (*red traces*). **(B)** Summary plot of the firing frequency (±SD) in response to current injections (from 0 to 250 pA) from SST-INs recorded in control ACSF and after PTX. **(C)** With-in cell comparison and mean (±SD) input resistance under baseline condition (CTRL) and in the presence of PTX. **(D)** The same as in **(C)** but for membrane potential. **(E)** The same as in **(C)** but for rheobase. **(F)** The same as in **(C)** but for maximal frequency. *p < 0.05, **p ≤ 0.01, and ***p ≤ 0.001.

Because L2/3 SST-INs had a low intensity of tonic inhibition (Figures 2, 3) we wondered if PTX application affected the intrinsic excitability of these interneurons (Figure 6). Similarly to VIP-INs, we observed a variety of SST-IN firing phenotypes, which were regular, low-threshold spiking with rebound spikes, accommodating without rebound spikes (Figure 6A and Supplementary Figure 1B), occasionally fast-spiking (Petilla Interneuron Nomenclature Group, 2008; Liguz-Lecznar et al., 2016; Urban-Ciecko and Barth, 2016). Here, the same as for VIP-INs, we pooled data from males and females for the analysis of excitability, because there were no differences in phasic and tonic inhibition of SST-INs in regards to the sex of animals (Figures 2–4). Surprisingly, for SST-INs we did not observe any differences in F-I curve between CTRL and after bath application of PTX (*n* = 7 cells, *p* > 0.05, two-tailed paired *t*-test, Figures 6A,B). Also, there were no changes in the input resistance (243.32 ± 53.37 MOhm in CTRL and 242.93 ± 37.06 MOhm in PTX, *n* = 7 cells in 7 mice, *p* = 0.578, Wilcoxon test, Figure 6C), the resting membrane potential (−49.77 ± 5.72 mV in CTRL and − 51.84 ± 7.71 mV in PTX, *n* = 7 cells in 7 mice, *p* = 0.479, two-tailed paired *t*-test, Figure 6D), rheobase (91.43 ± 21.16 pA in CTRL and 88.57 ± 18.64 pA in PTX, *n* = 7 cells in 7 mice, *p* = 0.457, two-tailed paired *t*-test, Figure 6E) and the maximal frequency in SST-INs (58.57 ± 20.32 Hz in CTRL and 48.86 ± 16.04 Hz in PTX, *n* = 7 cells in 7 mice, *p* = 0.136, two-tailed paired *t*-test, Figure 6F).

These data indicate that despite the presence of a low intensity of GABAAR-mediated tonic inhibition in L2/3 SST-INs, ambient GABA does not modulate the intrinsic excitability of these interneurons.

## Discussion

GABAARs regulate both fast synaptic (phasic) transmission as well as tonic (persistent) extrasynaptic inhibition (Farrant and Nusser, 2005; Glykys and Mody, 2007; Belelli et al., 2009). The tonic activation of GABAARs can regulate intrinsic excitability in a neuron-specific manner (Pavlov et al., 2009, 2014; Song et al., 2011). Here we investigated whether L2/3 SST-INs and VIP-INs might be regulated by tonic GABAAR inhibition. We compared tonic inhibition in interneurons to neighboring Pyr neurons, since tonic inhibition in Pyr cells has been studied before (Drasbek and Jensen, 2006; Vardya et al., 2008; Urban-Ciecko et al., 2010). Pharmacological blockade of GABAARs by PTX revealed tonic inhibition in both VIP- and SST-INs, however, the level of this inhibition was different in these cells. Namely, VIP-INs showed approximately two-fold stronger tonic inhibition than SST-INs. Surprisingly, tonic inhibition in SST-INs was comparable to Pyr cells in contrast to previous study, where a weak or no tonic inhibition in SST-INs has been found (Vardya et al., 2008; Donato et al., 2023).

Next, pharmacological activation of delta subunit-containing GABAARs by THIP showed that both interneuron types displayed tonic inhibition which was mediated by delta subunit and the level of delta subunit-induced tonic inhibition was around twice higher in L2/3 VIP-INs than in SST-INs. Thus, the differences in the values of tonic inhibition between VIP- and SST-INs might mainly come from the differential expression of the delta-subunit containing GABAARs in these interneurons. It has been found that hippocampal SST-INs show a very low expression of delta subunits and this is accompanied by a relatively low intensity of tonic current mediated by the delta-subunit containing GABAARs (Huang et al., 2023). Delta subunit-containing GABAARs have been found to be regulated by estrogens (Shen et al., 2005) and neurosteroids (Reddy and Kulkarni, 1999; Maguire et al., 2005; Wiltgen et al., 2005). Indeed, tonic inhibition mediated by these receptors was larger in L4 regular spiking (presumably Pyr neurons) and FS interneurons in the barrel cortex in females than in males (Urban-Ciecko and Mozrzymas, 2011). Nonetheless, in the present study there was no difference in the level of tonic inhibition between females and males. We hypothesize that the effect of the sex on tonic inhibition might be age-dependent because here mice were younger (P18-30) than in the prior work (P35-49) (Urban-Ciecko and Mozrzymas, 2011). Tonic inhibition can be mediated by GABAARs with other subunits. It has been found that alpha 5 subunit-containing GABAARs mediate tonic inhibition in L2/3 Pyr neurons but not in L2/3 PV-INs nor L2/3 SST-INs in the barrel cortex of X98 transgenic mouse line (Donato et al., 2023). Alpha 5-containing GABAARs have been revealed to be extrasynaptic, mainly mediating tonic inhibition (Caraiscos et al., 2004). However, alpha 5-containing GABAARs mediate also synaptic inhibition coming from X98 SST-INs to L2/3 Pyr cells but not to L1 interneurons of the barrel cortex (Donato et al., 2023). What is more, synaptic inhibition originating from VIP-INs to SST-INs is mediated by the receptors with this subunit in the hippocampus (Magnin et al., 2019) but not in the barrel cortex (Donato et al., 2023) indicating not only synapse- but brain area-specificity as well.

We also analyzed synaptic inhibition of both interneuron types and compared it to Pyr cells, because previous studies have reported that SST-INs in “GIN” mice possess minimal synaptic inhibition (Vardya et al., 2008). Here, we found that in fact sIPSCs had lower amplitude in VIP-INs than in SST-INs. In contrast, sIPSC frequency was comparable in both interneuron types. Also, sIPSCs were larger and more frequent in Pyr cells in comparison to VIP- and SST-INs, suggesting that synaptic inhibition is stronger in excitatory neurons than in these both interneuron types.

Finally, we observed that both the blockade and the activation of GABAARs had effects on the input resistance and the resting membrane potential suggesting that tonic inhibition might have essential effects on neuronal excitability. However, pharmacological blockade of GABAARs by PTX decreased the rheobase only in VIP-INs and had no effect on SST-INs, indicating that tonic GABAARs decrease intrinsic excitability of VIP-INs but not SST-INs. Altogether, our results indicate that GABAARs regulate interneuron excitability in a cell type-specific manner. L2/3 VIP-INs show relatively strong tonic inhibition and it reduces VIP-IN excitability, whereas L2/3 SST-INs display very weak tonic inhibition, which is unable to modulate the intrinsic excitability of SST-INs.

Literature data have shown that the effect of tonic inhibition on interneuron excitability is unclear and varies depending on the interneuron type (Pavlov et al., 2009, 2014; Song et al., 2011; Bryson et al., 2020). Surprisingly, depolarizing effect of GABA has been found in fast spiking (FS) CA3 stratum lucidum interneurons (Pavlov et al., 2014) and in other interneurons in the cerebellum (Chavas and Marty, 2003), amygdala (Woodruff et al., 2006) and striatum (Bracci and Panzeri, 2006). The combination of dynamic clamp experiments with neural network simulations has shown that the strength of tonic inhibition in the interneurons might control interneuron firing pattern and synchronization in the CA3 hippocampal network (Pavlov et al., 2014). Thus, an influence of tonic inhibition on the firing output of the interneurons is an essential factor for rhythm generation in the brain.

A separate observation in our study was that L2/3 VIP- and SST-INs displayed diverse spiking patterns in response to the somatic current injection in whole-cell patch-clamp recordings and that in fact these patterns were very similar in both interneuron types. In literature, the same firing patterns might have variety of terms (Kawaguchi and Kubota, 1996; Kawaguchi, 1997; Gibson et al., 1999; Beierlein et al., 2003; Goldberg et al., 2004; Wang et al., 2004; Ma et al., 2006; Prönneke et al., 2015, 2019; Liguz-Lecznar et al., 2016; Urban-Ciecko and Barth, 2016; Jiang et al., 2023). In the somatosensory cortex, the majority of SST-INs show so called classical accommodating (c-AC, Wang et al., 2004) spiking responses to current injection. This expression might be analogous to other terms such as regular spiking (RS) non-pyramidal (RSNP, Kawaguchi and Kubota, 1996; Kawaguchi, 1997) or low-threshold spiking in other studies (LTS, Gibson et al., 1999; Goldberg et al., 2004). In general, LTS interneurons are characterized by the relatively high input resistance, display accommodating pattern in response to depolarized currents and also generate rebound spike bursts following somatic hyperpolarization (Kawaguchi and Kubota, 1996; Goldberg et al., 2004). It has been revealed that not every SST-IN shows this phenomenon (Goldberg et al., 2004; Ma et al., 2006). In our experiments, SST-INs more often displayed LTS firing in the internal solution with the low [Cl^−^] concentration than in the high [Cl^−^] solution. As in other studies (Kawaguchi and Kubota, 1996; Wang et al., 2004; Ma et al., 2006), a small subgroup of SST-INs had a non-accommodating firing which was analogous to FS responses characteristic for PV (parvalbumin) interneurons. The same as for SST-INs, L2/3 VIP-INs might have firing patterns with regular spiking (adapting firing) as well as bursting adapting, bursting non-adapting or irregular spiking (Lee et al., 2010; Miyoshi et al., 2010; Prönneke et al., 2015, 2019; Jiang et al., 2023). In our experiments, a subset of VIP-INs exhibited high input resistance and displayed the classical LTS pattern with the rebound spikes. Similarly to SST-INs, LTS firing was observed more frequently in the internal solution with the low [Cl^−^] concentration.

Taking into account that L2/3 SST- and VIP-INs fire with very similar patterns, careful consideration should be given to studies using the firing phenotype as the only category to determine an interneuron type, since this population might include interneurons expressing different molecular markers. Now, thanks to a variety of transgenic mouse lines we can combine molecular and electrophysiological properties to study specific interneuron types. On top of that, recent investigations using single cell-PCR and multimodal classification methods differentiate several subgroups within VIP- and SST-INs (Gouwens et al., 2020; Donato et al., 2023; Hostetler et al., 2023; Jiang et al., 2023). Unfortunately, much of this diversity remains inaccessible for in-depth study due to lack of genetic targeting tools.

In conclusion, previous work has shown that ambient GABA was not sufficient to regulate L2/3 SST-IN excitability through GABABRs (Kanigowski et al., 2023). Here we demonstrate that GABAARs also do not regulate SST-IN firing, indicating that these interneurons are mainly immune to ambient GABA. In contrast, L2/3 VIP-INs are modulated by tonic GABAAR inhibition, as we report for the first time. Our results suggest that under the condition of ambient GABA, the differential sensitivity of VIP- and SST-INs to tonic GABAAR inhibition will favor SST-IN activity over VIP-INs. In this condition, SST-INs can generate powerful GABAergic inhibition thanks to their intrinsic excitability that is immune to both GABAA and GABABRs (Kanigowski et al., 2023).

Taken together, our data indicate that tonic GABAAR-mediated inhibition modulates neocortical networks in an interneuron type-specific manner. In overall, the differential sensitivity of specific neurons to GABA modulation may fine-tune the balance of excitation and inhibition in the cortical networks. Further studies are required to fully understand the complex role of tonic inhibition on neuronal network function under physiological and pathological conditions.

## Data availability statement

The original contributions presented in the study are included in the article/Supplementary material, further inquiries can be directed to the corresponding author.

## Ethics statement

The animal study was approved by Polish Ministry of Environment. The study was conducted in accordance with the local legislation and institutional requirements.

## Author contributions

KB: Formal Analysis, Investigation, Writing – original draft, Writing – review & editing. RK: Formal Analysis, Investigation, Writing – review & editing. JU-C: Formal Analysis, Investigation, Writing – review & editing, Conceptualization, Data curation, Funding acquisition, Methodology, Project administration, Resources, Supervision, Validation, Visualization, Writing – original draft.
