# Supplementary figures and images for: GABAAR-mediated tonic inhibition differentially modulates intrinsic excitability of VIP- and SST- expressing interneurons in layers 2/3 of the somatosensory cortex
Source: Front Cell Neurosci. 2023 Oct 12;17:1270219. doi: 10.3389/fncel.2023.1270219 (PMC10602639; doi:10.3389/fncel.2023.1270219)

## Slide 1
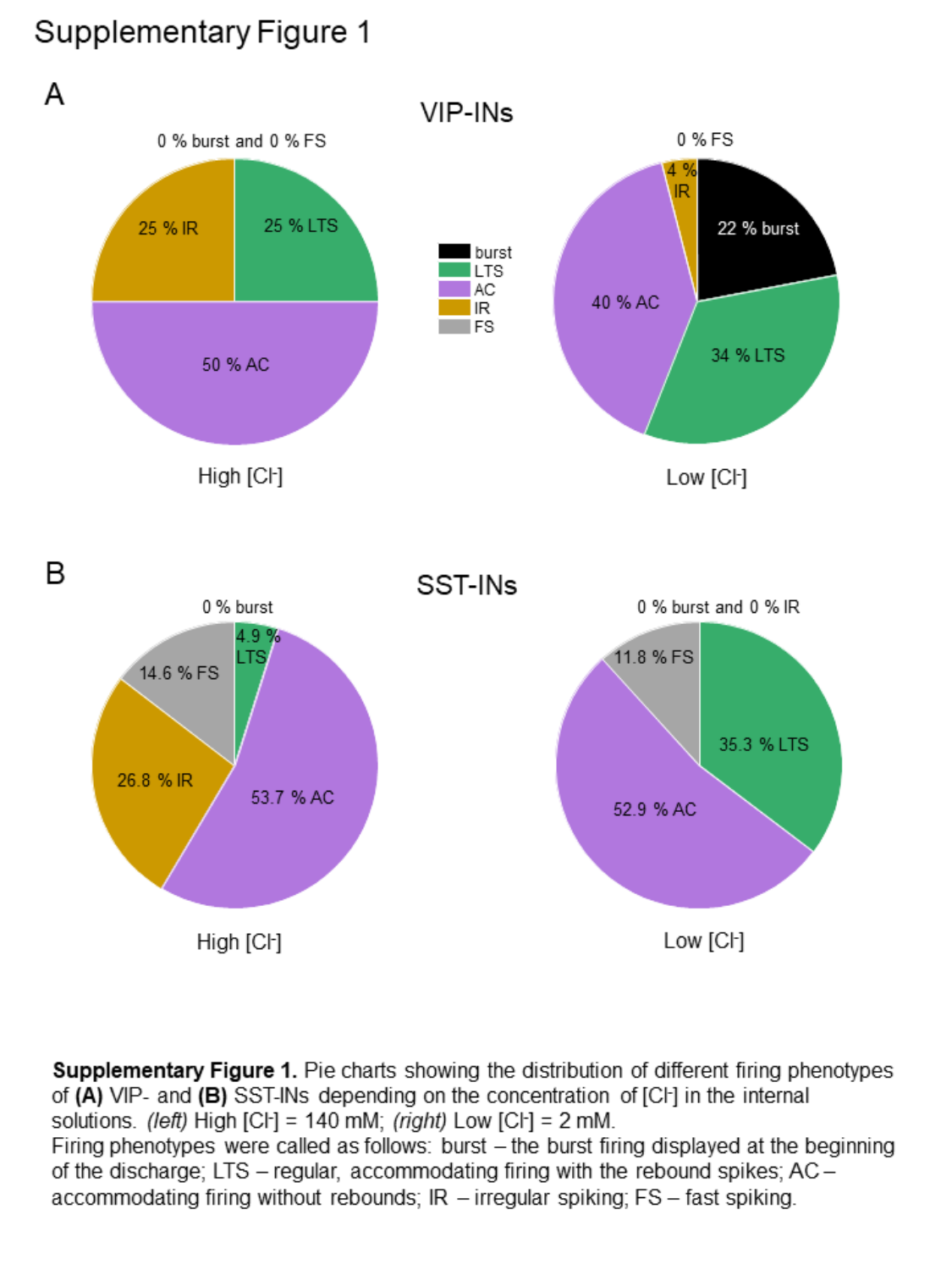

## Slide 2
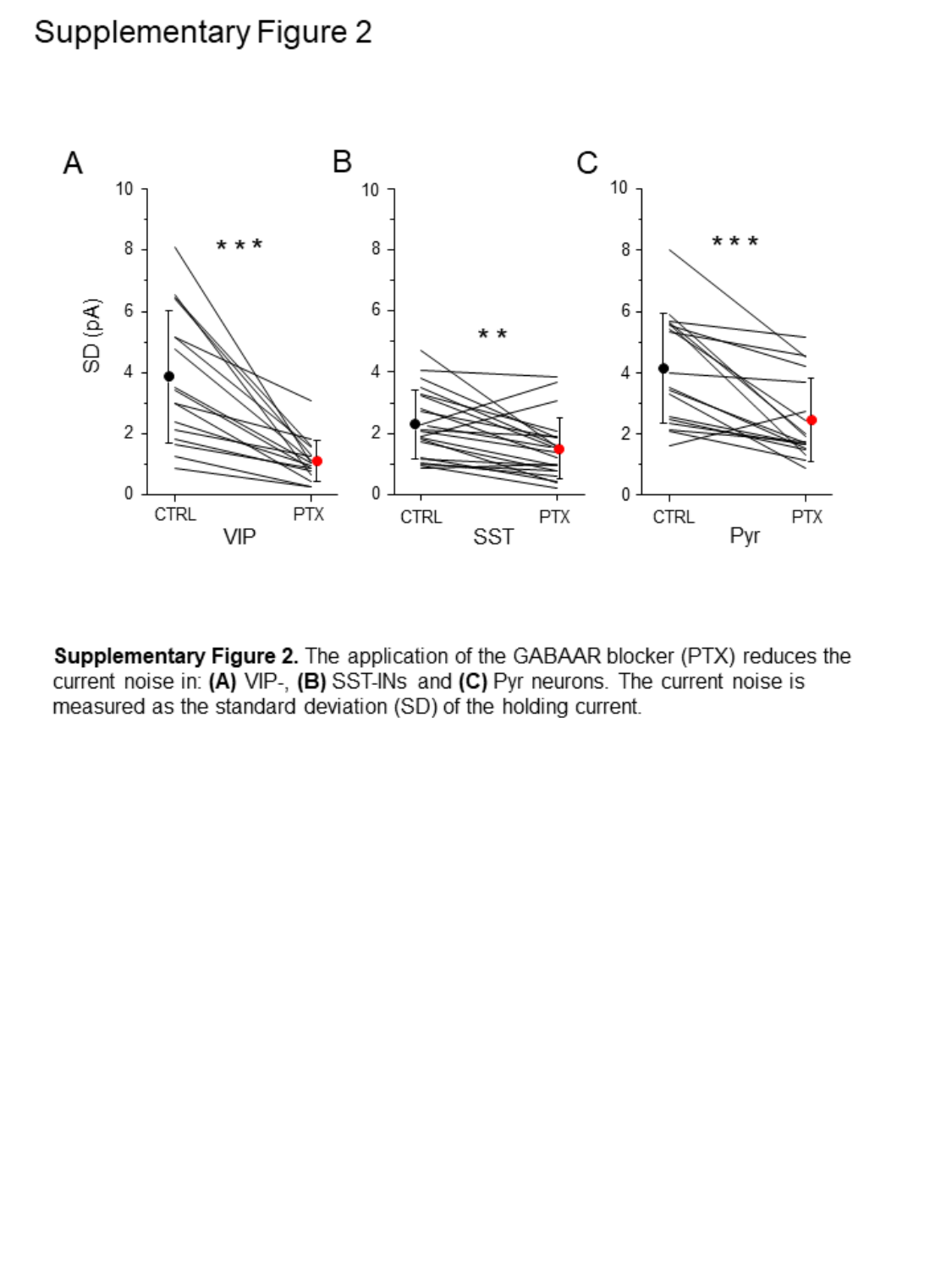

## Slide 3
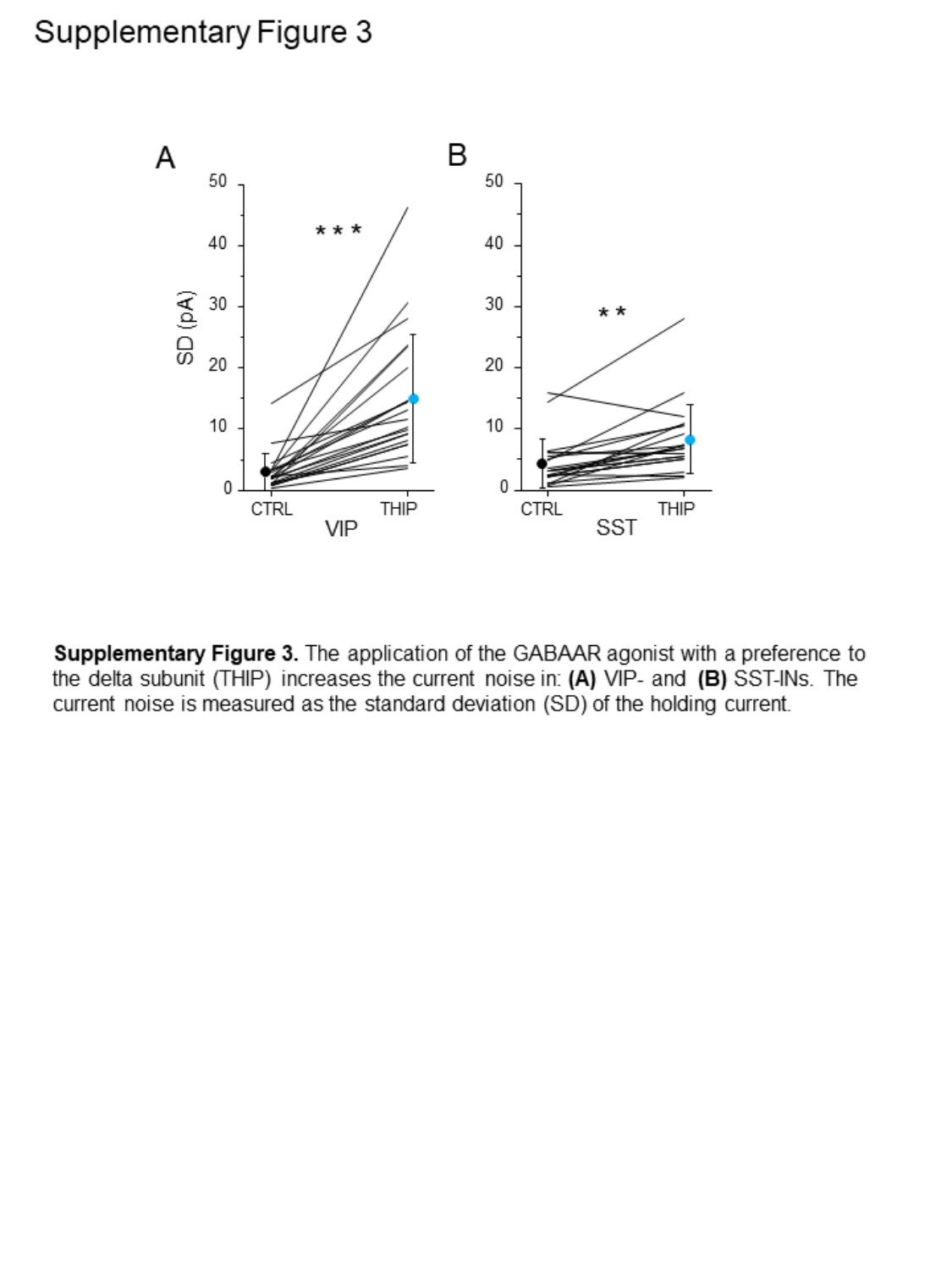

Supplement: Supplementary file 1 [file Presentation_1.PPTX]
